# Medication Noncompliance among Patients with Chronic Diseases Attending a Primary Health Facility in a Periurban District in Ghana

**DOI:** 10.1155/2018/7187284

**Published:** 2018-06-07

**Authors:** Bright Addo, Sally Sencherey, Michael N. K. Babayara

**Affiliations:** ^1^University of Ghana School of Public Health, Legon, Accra, Ghana; ^2^Komfo Anokye Teaching Hospital (KATH), Kumasi, Ghana; ^3^Postgraduate College, 37 Military Hospital, Accra Central, Ghana

## Abstract

**Background:**

Despite the growing interest in understanding the aetiology of chronic diseases, limited studies exist on medication noncompliance, especially, among periurban and rural dwellers in Ghana. In this study, we determined the prevalence of medication noncompliance and explored the medication intake behaviour of patients with chronic diseases. The relative influence of cost on medication noncompliance and the risk factors for noncompliance were also assessed.

**Methods:**

The design was a cross-sectional study of 200 patients from ages below 40 years to ages above 60 years sampled from the Offinso South Municipality, a periurban district of the Ashanti region of Ghana. Data collected through the administration of structured questionnaires was coded, cleaned, and analysed using the SPSS (v20) software programme. Descriptive and multivariate analyses using binary logistic regression were performed.

**Results:**

Medication noncompliance was high (55.5%), with patients living with HIV/AIDS and those with psychological disorders being the most noncompliant. Majority of patients took at least 2 medications (81.5%), did so twice daily (79.0%), did not experience side effects with intake (67.0%), considered their medication to be effective (88.5%), and were aware of the complications that could arise from noncompliance. The dominant route of medication intake was oral (86.8%) and a lesser proportion of patients (22.5%) took herbal preparation alongside their prescribed medications. The cost of medication did not prevent patients from adhering to their medication regimen as most of these drugs were covered by the National Health Insurance Scheme (NHIS). Age, duration of diagnosis and difficulty in remembering medication instructions were identified as significant predictors of noncompliance.

**Conclusion:**

Educating patients on the need to be compliant with their medication regimen, the complications that could arise from noncompliance and avoidance of intake of herbal medications during their treatment should form part of the clinical sessions organized for patients with chronic conditions.

## 1. Introduction

The success of every therapeutic regimen depends on the compliance of the individual involved. The efforts put in by healthcare providers can therefore only yield the desired effect if patients are compliant to their medication regimen. Unfortunately, medication noncompliance with its associated detrimental effects is becoming widespread and has been found to be most prevalent among patients with chronic diseases.

Globally, chronic diseases have been found to be the leading cause of mortality and disability, and the disease rates from these conditions are not only accelerating but advancing across every region and pervading every socioeconomic class [1]. The WHO projects that, by 2020, the incidence of chronic disease will account for almost three-quarters of all death occurring worldwide, and that 71% of deaths due to ischaemic heart disease (IHD), 75% of deaths due to stroke, and 70% of deaths due to diabetes will occur in the developing countries [2]. The WHO further estimates that the number of people in the developing world with diabetes will increase by more than 2.5-fold, with the 1995 number of 84 million sharply rising to 228 million in 2025. On a global scale, 60% of the burden of chronic diseases and 79% of deaths attributed to these diseases are expected to occur in developing countries, of which Ghana is included [2].

In Ghana, hypertension alone is said to have accounted for between 19% and 54.8% of the outpatient morbidity in adults above 45 years in 2012 [3]. Diabetes mellitus also accounted for 6.8% of all adult admissions and also 7.8% of all adult deaths in at the Korle-Bu Teaching Hospital [4].

Among patients with chronic diseases such as hypertension, diabetes, HIV/AIDS, and psychiatric illnesses, medication noncompliance has been found to be very common [5, 6].

Poor adherence to therapy among hypertensive patients contributes to two-thirds of poor blood pressure control [5]. Failure to achieve blood pressure control subsequently leads to development of cardiovascular complications including myocardial infarction and stroke. For patients with psychiatric disorders, not only do they have a challenge following a medical regimen, but they also have the greatest potential for benefiting from compliance [7].

Among patients living with HIV/AIDS, with the advent of antiretroviral drugs, anything less than thorough compliance can result in reduced efficacy of the drugs and later lead to development of resistant viral strains [8].

There is a threefold effect to medication noncompliance, and these effects are manifested in the clinical outcome of the patient, the cost of treatment, and the risk of hospitalizations, all of which have been identified as the main cause of failure to effectively manage chronic diseases [9–11].

Over the last two decades, there have been a plethora of studies that have examined variables that could be demonstrated as predictive of adherence to various medical regimens. The factors most often hypothesized in these studies as powerfully predicting compliance have generally been attributed to characteristics of both the disease and the patients. For example, to explore and evaluate the most common factors causing therapeutic noncompliance, Jin and colleagues found factors that could be categorized into (1) patient-centered factors, (2) therapy-related factors, (3) social and economic factors, (4) healthcare system factors, and (5) disease factors [9]. Factors which relate to patients (e.g., suboptimal health literacy and lack of involvement in the treatment decision-making process), physicians (e.g., prescription of complex drug regimens, communication barriers, ineffective communication of information about adverse effects, and provision of care by multiple physicians), and health care systems (e.g., limited access to care, lack of health information technology, and office visit times limitations) have also been found [12].

In the West African subregion and particularly in Ghana, studies have identified specific factors such as depression, concern about disease medications, formal education, and use of herbal preparations to be associated with nonadherence among hypertensive patients [11] and factors such as educational level and mode of payment to be associated with nonadherence among diabetic patients [10].

In an era where cost-effectiveness is a buzz word in healthcare delivery, these identified factors must be a concern for healthcare providers and health systems [9]. Unfortunately, risk factor studies identifying the proximate determinants of medication noncompliance have generally been inconclusive. Whiles some factors have been identified to significantly associate with noncompliance in one geographical context, same factors have been found to be nonsignificant in others.

With the persistent rise in the prevalence of chronic diseases, medication compliance as an area of research study is gaining much recognition and whiles there is now ample research on the subject, most of these studies have been conducted in developed countries. The extant literatures on medication nonadherence in Ghana have mostly focused on the two chronic diseases, hypertension and diabetes much to the neglect of other chronic conditions such as HIV/AIDS and psychiatric disorders. The plethora of studies which even do exist on medication nonadherence among diabetic and hypertensive patients have mostly been conducted in urban settings of the country, with limited studies conducted among periurban and rural dwellers. On this premise, this study sought to determine the prevalence of medication noncompliance among patients with chronic diseases in a periurban district in Ghana. Medication intake behaviour, the relative influence of cost on medication noncompliance, and the risk factors for medication noncompliance were also assessed.

## 2. Methods

### 2.1. Study Area

The study was conducted in the Offinso South Municipality, one of the 30 Municipals in the Ashanti region of Ghana. The 2010 Population and Housing Census put the population of the municipality at 76,895 with a population density of 131 persons per square kilometre. A high percentage (70.0%) of the population in the municipality is economically active, with over 50.0% of the employed being skilled agricultural and fishery workers [13]. More females (16,071) than males (14,348), of the working age population in the municipality, have an occupation. On health, the Municipality Health Services are organized around 5 submunicipalities: namely, Bonsua, Abofour, Offinso Central, Anyinasuso, and Kwagyekrom. There are eleven (11) health facilities with the ownership being government/public, CHAG, and private. Due to the absence of a municipal government or public hospital, St. Patrick's Hospital is earmarked as the municipal hospital. In addition, there are 3 functional CHPS compounds and 42 CHPS zones with the respective CHOs assigned. There are also 76 trained TBAs and 61 CBSVs. The municipality recorded a total of 455 household deaths 12 months prior to the 2010 PHC representing 1.6% of the entire Ashanti region. The crude death rate, which is the number of deaths per 1000 of the population in the Municipality, is 5.9 [13].

### 2.2. Study Design

This was a cross-sectional descriptive survey using systematic random sampling to collect quantitative data involving multiple variables which were analysed to determine distribution patterns and test relationships.

### 2.3. Study Population

The sampling population included people aged 18 and above presenting at the special OPD clinic at St. Patrick's Hospital who had been diagnosed of a chronic ailment and consented to participate in the study.

#### 2.3.1. Inclusion Criteria

To be included in the study, patients had to be diagnosed of at least one out of the four chronic diseases of interest, that is, hypertension, diabetes, HIV/AIDS, or psychiatric disorder. The patient should also have been on medication for more than 6 months and must have consented to participate in the study.

#### 2.3.2. Exclusion Criteria

Patients who had been diagnosed of any of the chronic diseases of interest and were yet to start medication or had been on medication for less than 6 months were exempted from the study. Patients below the age of 18 years were also excluded.

### 2.4. Sample and Sampling Technique

The hospital facility was chosen for the study as it was easier to get access to patients that met the inclusion category. Of the number of health facilities available at the Offinso South Municipality, St. Patrick's Hospital was purposively selected as it was the largest hospital and was frequented by most people in the municipality.

The systematic random sampling technique was used to sample 200 eligible patients. At a particular clinic, the first person was given the chance to participate and every third person was also sampled to participate. Consent was sought from the selected participant before inclusion into the study.

### 2.5. Instrumentation

Standard questionnaires were used for the study. The study's research questions and objectives informed the design of the questionnaires. Prior to the design, a thorough literature search was conducted to determine and categorize concepts and variables used in studies related to the topic. Information from the literature review focused on issues relating to noncompliance to medication regimen among patient with chronic diseases. The research instrument crafted for this study was a 28-item self-reporting research instrument utilizing closed ended questions with response categories that were precoded which facilitated numerical coding of the data after collection. The entire questionnaire was arranged into content subsections A, B, and C.

Section “A” consisted of nine (9) sociodemographic survey items which distinguished patients in terms of their age, gender, religion, employment status, occupation, marital status, educational attainment, chronic condition(s) suffered, and duration of condition since diagnosis. Section “B” consisted of 14 questionnaire items developed to measure patients' compliance to medication regimen. The questions asked mainly bothered on the different medications patients took for their condition, frequency of daily intake, route of administration of medication, number of tablets/capsules taken daily, adherence to doctor's instructions as to how medication should be taken, side effects experienced if any, etc. (see attached study questionnaire for full details available here).

Section “C” also consisted of 4 questions developed to measure the influence of cost on compliance to medication. The first question ascertained whether patients' medication was covered by the National Health Insurance Scheme (NHIS), whiles the second was a follow-up question for respondents who indicated “No” as a response to indicate how they paid for their drugs. The third and fourth questions requested patients to indicate the cost of their medication(s) and whether they missed their medication for a period of time due to cost.

Additionally, there were standard instructions that requested respondents to select the most suitable answer with the assurance that there was no right or wrong answer in the selection of answers to the questions.

### 2.6. Validation and Pretesting of Questionnaire

The questionnaire validation process was a two-step one. The first step was the establishment of face validity of the questionnaire which was achieved by consulting two experts: (1) a university professor and (2) a practicing physician. These two experts were tasked to ascertain if the questions asked effectively captured the topic under investigation. Their suggestions and recommendations made were then incorporated.

A pretest was then carried out after the validation to obtain information to improve the questionnaire and to assess the feasibility of the study. The respondents in the pretest were similar to those in the study and it was carried out under similar settings. Conducting the pretest helped to identify problems with the questionnaire, to gauge the time needed to complete the questionnaire and ultimately, and to ensure that the questions were understood by respondents. The feedback obtained helped in fine-tuning the questionnaire. The pretest was conducted among 15 patients at the Komfo Anokye Teaching Hospital (KATH).

### 2.7. Data Collection

Data was collected over a period of one month, from March to April, 2016 at the St. Patrick's Hospital which doubles as the municipal's hospital after pretesting of the research questionnaire. Quantitative data was primarily sought for the study. The data was obtained through the use of a structured questionnaire specifically designed to suit respondents understanding of the study. The response categories of the various questions or variables were mostly precoded. A word of acknowledgment was rendered to study respondents for their participation in the survey.

### 2.8. Data Analysis

The data collected was first edited to ensure that all questionnaires were complete and properly filled. In all, 200 complete questionnaires were obtained from the respondents. After editing, the data from the completed questionnaire was coded and entered into the SPSS (v20) programme. Preliminary data analysis was conducted to obtain frequency distribution for all variables. The preliminary analysis also served as a cleaning strategy which helped to identify data entry errors. The validation tool in the SPSS programme which gives information about missing values with their identification numbers and wrong entries made was further utilized to confirm that the data was clean and ready for analysis.

Descriptive analysis was conducted on respondents' background characteristics and reported in frequencies and percentages. Multivariate analysis using binary logistic regression was conducted to determine the factors for noncompliance to medication regimen.

### 2.9. Ethical Considerations

Ethical approval was obtained from the Ethical Review Committee of the Komfo Anokye Teaching Hospital (KATH) through the School of Medical Sciences, KNUST, Kumasi. Permission for the research was granted from the Administration of St. Patrick's Hospital as well as the head of the special clinic of the hospital. The purpose of the study was explained to every participant. Confidentiality was assured and either verbal or written consent was sought from every individual who participated in the study.

Participation in the study was not compulsory and anonymity of respondents was respected. All respondents voluntarily gave verbal consent to participate in the study after the rationale of the study was explained to them. Couples were interviewed separately and care was taken to ensure privacy and confidentiality.

## 3. Results

### 3.1. Background Characteristics of Respondents

A total of 200 patients participated in the study. Patients' ages ranged from below 40 to 60+ years with majority of them being less than 40 years and between 40 and 60 years. Together, these two age cohorts constituted 79% of the entire sample size. Majority of the respondents were females (76%), were in marital unions (57%), had attained basic education, that is primary and up to Junior High School (59%), were into trading, and belonged to the Christian faith (88%). Patients who were diagnosed with diabetes were the most (42.4%), followed by those with hypertension and HIV/AIDS. Patients who had lived with their disease condition between 1 and 5 years constituted a half (which was majority) of the entire sample (see Table 1).

### 3.2. Prevalence of Medication Noncompliance

**Figure 1:**
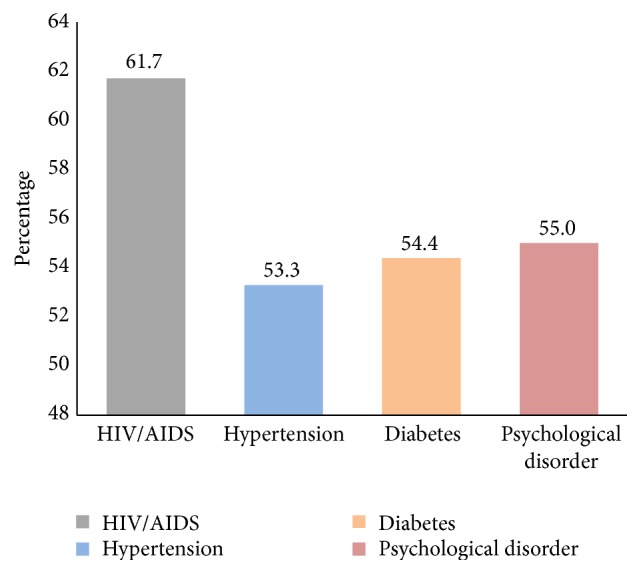
Proportion of patients with chronic diseases who are noncompliant to their medication regimen.

Medication noncompliance (assessed by patients indicating whether they always admitted to their doctor's instruction) in the sample of 200 patients was found to be relatively high (*n* = 111, 55.5%) (Table 2(a)). Comparatively, patients with HIV/AIDS and those with psychological disorders were found to be the most noncompliant (61.7%; 55.0%, respectively). Patients who were hypertensive were the least noncompliant (53.3%) (Figure 1).

### 3.3. Medication Intake Behaviour of Patients

Majority of the patients (81.5%) were found to be taking at least 2 medications. Patients who were on exactly two medications for their disease condition were however the most (38.0%). A higher percentage of the patients (79.0%) took their medication twice daily, with the dominant route of intake being orally (86.8%). More than half (55.8%) of the patients who were on oral medications reported they took less than 5 tablets daily. Majority of the patients (67.0%) did not experience side effects with the intake of their drugs and the few who did (33.0%) mostly complained of general body weakness and insomnia. Of those who experienced side effects, most were able to tolerate it (Table 2(a)).

A lesser proportion of patients (22.5%) took herbal preparation alongside their prescribed medications and out of that, 8.0% stopped taking their prescribed medications and 8.5% took it alongside prescribed medications, which is an unhealthy practice. Majority of the patients (93.5%) had no problem with remembering medication instructions and forgetfulness was a problem in only about 16.0%. A higher percentage of the patients considered their medication effective (88.5%), were aware of the complications that could arise from noncompliance (58.0%), and were self-reminded to take their medications (87.5%) (see Table 2(b)).

### 3.4. Cost and Noncompliance to Medication Regimen

Most of the drugs patients used (71.0%) were covered by the NHIS. Out of the 29.0% of patients who had to pay for their drugs, 15.5% paid from their own income and 13.5% were being supported by family members; however, 12% were missing their medications because they could not at a point in time pay for their drugs (Table 3). The cost of drugs patients had to purchase by themselves mostly ranged from between 10 and 30 Ghana cedis ($2.22–$7.5).

### 3.5. Factors for Medication Noncompliance

Based on the three main categories of factors (i.e., personality characteristics and disease related and medication related factors) identified in the literature to influence medication compliance, the regression analysis was modelled to contain the variables comprising these three factors. Of the twelve factors (4 personality characteristics and 1 disease related and 7 medication related factors) entered into the regression model, only age, duration of diagnosis, and difficulty remembering medication instructions were found to be significant predictors of noncompliance to medication regimen. Patients who were old were more likely to be noncompliant. Those whose disease condition was long diagnosed were found to be 2 times more likely to be noncompliant; likewise those who did not have difficulty remembering medication instructions are 6 times more likely to be noncompliant (see Table 4).

## 4. Discussion

There has been a dearth in studies to assess the level of noncompliance to medications for chronic diseases over the years, especially among patients with chronic conditions living in rural and periurban districts in sub-Saharan Africa. Taking cognisance of the fact that the success of every therapeutic regimen depends on the compliance of the individual involved and that medication noncompliance is becoming widespread, we found this study to be crucial and timely. We found the overall prevalence of medication noncompliance among the patients to be 55.5%. This rate is higher than that of a similar study conducted in Northwest Ethiopia (42%) to determine noncompliance in patients with chronic illnesses [14]. The highest level of compliance and in effect the lowest level of noncompliance, was recorded among patients with hypertension, 53.3%. This percentage in hypertension is close to the level, 52.3% obtained in another study at the Korle-Bu Teaching hospital [6] and lower than 93% recorded in a similar study at KATH [15].

We found compliance to antihypertensive drugs to be comparatively better probably because there has been much more education on hypertension leading to interventions to improve compliance among patients with hypertension. The reduced side effects and the NHIS covering some hypertensive medications could also be a contributing factor. Among the four disease conditions, we found the prevalence of medication noncompliance to be the highest among patients living with HIV/AIDS (61.7%). This rate is high compared to a study conducted in Eldoret which revealed a noncompliance prevalence rate of 36.8% [8]. This high rate in noncompliance could be attributed to the stigma attached to the disease. Some patients affected by the disease have to travel distant towns for their medication in order not to be seen by people. They can therefore delay in getting their medications refilled if they are unable to make it to these distant towns. Added to this is the fact that some of these patients have to hide in order to take their medications so that the people around them do not get suspicious of an ailment. This implies that in situations where there is no privacy these patients may miss their medications.

The level of nonadherence to medications for diabetes in our study was found to be 54.4%. Comparatively, the rate is higher than that obtained in a Ugandan study which recorded a prevalence rate of 28.9% nonadherence among patients [16]. Another study in Nigeria however showed a much higher level of nonadherence, 73.6% [17]. The level of noncompliance among patients with psychiatric illnesses was found to be 55.0%. This was the overall level of adherence of the patients reporting at the psychiatric clinic, some of whom had mainly schizophrenia, bipolar disorders, and epilepsy.

Our study reports a medication intake behaviour where a high proportion of patients were on oral medications which was taken twice daily and a lesser proportion taking herbal preparations alongside their prescribed medications. Some patients however stopped the intake of their prescribed medications and resorted to the use of herbal drugs, a practice we find to be unhealthy. There has been a current upsurge in the use of herbal medication to treat chronic diseases in Ghana, especially diabetes. This upsurge has been fueled by the several advertisements aired by most of the mass media houses in the country. Often times, the efficacy of the drugs being advertised is yet to be scientifically proven, though most claim to have received an FDA approval.

On the effect of medication cost on noncompliance, we found that patients who had all or some of their medications covered by the NHIS were generally more compliant. The cost of medications, therefore, to some extent has the potential of influencing patients' compliance which implies that having more medications covered by the NHIS can improve overall adherence. This finding is consistent with the study of Buabeng and colleagues conducted at the Korle-Bu Teaching Hospital (KBTH) in Ghana which found unaffordable drug prices as the major cause of noncompliance among patients with hypertension [15]. Medications such as insulin are not covered by the NHIS, and with the cost of the drug being high, majority of patients could miss their medications because they cannot afford to purchase them.

Variations in the significant factors predicting medication compliance and noncompliance have been observed in numerous studies. While some factors have been found to be significant in some studies, the same factors have been found to be nonsignificant in other studies. In this study we found patients age, duration of diagnosis, and difficulty remembering medication to be the significant predictors of medication noncompliance. Consistent with the study of Boima and colleagues, we also found the elderly (old) to be less compliant than the young. This pattern observed in noncompliance has also been documented in studies carried out in Sub-Saharan Africa [11].

Patients whose disease condition had persisted over a longer period of time were found to be 2 times more likely to be noncompliant. This finding is similar to that of Hyre and colleagues who also found that a longer duration of disease conditions may not only compromise patients' compliance but also affect compliance negatively [18]. A plausible reason for this could be the fear patients have initially when they are diagnosed with an ailment. With time, they see it as a norm and are no longer afraid of the complications that could arise with their default. Also after taking medications for a long time, patients get worn-out and seek for a somewhat permanent cure, the most likely option being resorting to herbal medications which promise a pull-up of the underlying disease cause. In the descriptive analysis we found that majority of the patients did not have difficulty remembering their medication instructions, in the regression analysis; however, we found that patients who did not have difficulty remembering their medication instruction were however 6 times more likely to be noncompliant. A possible explanation to this observation could be due to the fact that once patients understand and have become familiar with their intake routine they could choose to alter it, especially when they begin experiencing some side effects.

## 5. Limitations

Our study was limited to patients receiving health care at the St. Patrick's Hospital and did not include patients who attended other health centers in the municipality though they might have met the inclusion criteria. This limitation notwithstanding, we anticipate that since the hospital serves as a primary health facility in the municipal, it records majority of the OPD cases and as a result we may have been able to capture most of the patients with chronic conditions. Additionally, engaging in direct observation of clients and pill counting could have made the study findings more robust; however, due to the laboriousness of this methodology it could not be adopted. Despite these limitations, the findings of our study are encouraged.

## 6. Conclusion

Noncompliance to medication regimen among chronic disease patients is an important issue for public health consideration. This is evidenced by the results of this study which recorded an overall noncompliance level of 55.5%. The factors identified as contributing to medication noncompliance in this study were age, duration of disease condition, and difficulty in remembering medication instructions. To reduce the incidence of medication noncompliance among chronic disease patients, we first recommend that programmes be drawn in the various clinics for the purpose of health education and counselling of the patients during their waiting times with emphasis on the importance of their medications and the consequences of nonadherence. Emphasis must also be placed on the detrimental effects of mixing orthodox medications with herbal preparations. Opportunity should be created at the clinics during the waiting times for patient-to-patient interaction to share their experiences and challenges so that they could encourage themselves through the difficulties of their therapies. Doctor-to-patient relationship must also be improved so patients can freely discuss issues relating to their therapy. The government of Ghana, through the Ministry of Health, could be sought to extend the NHIS to cover more of the medications used by chronic disease patients. This would reduce the financial burden of patients on life-long therapies.

## Figures and Tables

**Table 1:** Distribution of respondents by background characteristics (*N* = 200).

Characteristic	Frequency (*n*)	Percent (%)
Age (years)		
** **<40	80	40.0
** **40–60	79	39.5
** **>60	41	20.5
Gender		
** **Male	48	24.0
** **Female	152	76.0
Religion		
** **Christian	176	88.0
** **Islam	16	8.0
** **African Traditional	4	2.0
** **None	4	2.0
Occupation		
** **Farming	41	20.5
** **Trading	68	34.0
** **Artisan	13	6.5
** **Teacher	5	2.5
** **Driver	6	3.0
** **Health worker	1	.5
** **Mechanic	1	.5
** **Unemployed	65	32.5
Marital status		
** **Single	43	21.5
** **Married	114	57.0
** **Divorced	14	7.0
** **Widowed	29	14.5
Educational attainment		
** **None	45	22.5
** **Primary	40	20.0
** **Junior High School	77	38.5
** **Senior High School	28	14.0
** **Tertiary	10	5.0
Chronic diseases suffered^a^		
** **HIV/AIDS	60	22.3
** **Hypertension	75	27.9
** **Diabetes	114	42.4
** **Psychiatric illness	20	7.4
Duration of diagnosis		
** **Less than a year ago	15	7.5
** **1–5 years ago	100	50.0
** **5–10 years ago	38	19.0
** **More than 10 years ago	47	23.5

^a^Multiple response item.

**Table:** (a) Medication intake behaviour of patients (*N* = 200)

Characteristic	Frequency (*n*)	Percent (%)
Number of medications taken for condition		
** **One	25	12.5
** **Two	76	38.0
** **Three	43	21.5
** **Four	36	18.0
** **More than 4	20	10.0
Frequency of intake		
** **Once daily	28	14.0
** **Twice daily	158	79.0
** **Thrice daily	14	7.0
Route of intake^a^		
** **Oral	197	86.8
** **Injection	30	13.2
Number of tablets taken if oral		
** **Less than 5	110	55.8
** **Between 5 and 10	82	41.6
** **More than 10	5	2.6
Always admit to doctor's instructions		
** **Yes	89	44.5
** **No	111	55.5
Notification of side effects of drugs		
** **Yes	66	33.0
** **No	134	67.0
Side effects mostly experienced^c^		
** **Weakness	13	6.5
** **Insomnia	8	4.0
** **Dizziness	6	3.0
** **General malaise	6	3.0
** **Irritability	4	2.0
** **Lethargy	4	2.0
** **Oversleeping	4	2.0
** **N/A^b^	134	67.0
Able to tolerate side effects		
** **Yes	40	20.0
** **No	26	13.0
** **N/A^b^	134	67.0

^a^Multiple response;  ^b^N/A = patients who indicated that they did not experience any side effects;  ^c^predominant side effects experienced by patients.

**Table:** (b) Medication intake behaviour of patients (*N* = 200)

Characteristic	Frequency (*n*)	Percent (%)
Use of herbal medication		
** **Yes	45	22.5
** **No	155	77.5
Influence of intake on medication compliance		
** **Doesn't influence it	17	8.5
** **Stop taking the prescribed medication	16	8.0
** **Take it alongside prescribed medication	12	8.0
** **N/A^a^	155	77.5
Difficulty in remembering medication instructions		
** **Yes	13	6.5
** **No	187	93.5
Medication is effective		
** **Yes	177	88.5
** **No	10	5.0
** **Sometimes	13	6.5
Frequency of forgetting to take medications		
** **Daily	3	1.5
** **Frequently	28	14.0
** **Rarely	104	52.0
** **Never	65	32.5
Awareness of complications arising from non-compliance		
** **Yes	116	58.0
** **No	84	42.0
Ways of being reminded to be compliant with medications		
** **Doctor's advice	5	2.5
** **Advice from other health workers	2	1.0
** **Radio or TV	1	.5
** **Friends or family	17	8.5
** **Self	175	87.5

^a^N/A = patients who indicated that they did not use herbal medication.

**Table 3:** Cost and medication noncompliance (*N* = 200).

Variable	Frequency (*n*)	Percent (%)
Medication covered by NHIS		
** **Yes	142	71.0
** **No	4	2.0
** **Some of them	54	27.0
Means of paying for drugs		
** **From income	31	15.5
** **Support of family members	27	13.5
** **N/A^a^	142	71.0
Cost of medications		
** **Less than 10 cedis	7	3.5
** **10–30 cedis	33	16.5
** **31–60 cedis	15	7.5
** **61+ cedis	3	1.5
** **N/A^a^	142	71.0
Missed medication because of cost		
** **Yes	24	12.0
** **No	34	17.0
** **N/A^a^	142	71.0

^a^N/A = not applicable.

**Table 4:** Multivariate analysis of risk factors for medication noncompliance.

Factor	B	S.E.	*p*-value	OR (95% CI)
Gender				
Male	1			
Female	−.335	.394	.395	.715 (.331–1.547)
Age				
Young	1			
Old	−1.047	.384	.006^*∗*^	.351 (.165–.745)
Education				
Not educated	1			
Educated	−.567	.411	.168	.567 (.253–1.270)
Marital status				
Single	1			
Married	.137	.324	.673	1.147 (.608–2.163)
Duration of diagnosis				
Short (<6 years)	1			
Long (>5 years)	.794	.336	.018^*∗*^	2.213 (1.145–4.278)
Number of tablets taken orally				
Less than 5	1			
Between 5 and 10	.452	.368	.219	1.571 (.764–3.232)
More than 10	−.213	1.096	.846	.808 (.094–6.919)
Notification of drugs side effects				
Yes	1			
No	.304	.335	.363	1.356 (.704–2.612)
Use of herbal medication				
Yes	1			
No	−.230	.387	.552	.794 (.372–1.697)
Difficulty remembering medication instructions				
Yes	1			
No	1.715	.736	.020^*∗*^	5.557 (1.313–23.524)
Efficacy of medication				
Yes	1			
No	.323	.767	.674	1.381 (.307–6.217)
Sometimes	1.304	.793	.100	3.685 (.779–17.433)
Awareness of complications that can arise from non-compliance				
Yes	1			
No	.313	.324	.334	1.367 (.724–2.580)
Medication covered by NHIS				
Yes	1			
No	−.717	1.176	.542	.488 (.049–4.893)
Some of them	.743	.363	.041	2.102 (1.032–4.278)

^*∗*^*p* < .05.

## Data Availability

The study questionnaire used for collection of data is attached as a supplementary file. The dataset used can also be obtained from the corresponding author on reasonable request.

## Abbreviations

CHPS: Community-Based Health Planning and Services
GDHS: Ghana Demographic Health Survey
GHS: Ghana Health Service
NHIS: National Health Insurance Scheme.

## Supplementary Materials

Supplementary Materials Study Questionnaire: medication noncompliance in patients with chronic diseases in the Offinso South Municipality.
