# Supplementary material for: Medication Noncompliance among Patients with Chronic Diseases Attending a Primary Health Facility in a Periurban District in Ghana
Source: Int J Chronic Dis. 2018 Jun 7;2018:7187284. doi: 10.1155/2018/7187284 (PMC6011085; doi:10.1155/2018/7187284)
Supplement: Supplementary Materials — Study Questionnaire: medication noncompliance in patients with chronic diseases in the Offinso South Municipality. [file 7187284.f1.docx]

**MEDICATION NON-COMPLIANCE IN PATIENTS WITH CHRONIC DISEASES IN THE OFFINSO SOUTH MUNICIPALITY**

This study is to assess the reasons for medication non-compliance among patients with chronic diseases in the Offinso South Municipal. Your participation is voluntary. All information given is anonymous and confidential. It will be greatly appreciated if you would kindly provide the required information and give honest responses to the questions posed. Thank you for your cooperation.

A. Demographic details

1. Age
2. Below 40 { } 2. Between 40 and 60{ } 3. More than 60{ }
3. Gender
4. Male { } 2. Female{ }
5. Religion
6. Christian { } 2. Islam { } 3. African traditional religion { } 4. Others – Specify _________________
7. Employment Status: 1. Employed [ ] 2. Unemployed [ ]
8. Occupation: _______________________
9. Marital status

1. Single 2. Married { } 3.Divorced { } 4.Widowed { }

1. Educational Background:
2. None 2.Primary{ } 3.JHS{ } 4.SHS{ } 5..Tertiary{ }
3. What chronic condition(s) do you have?
4. HIV/AIDS { } 2. Hypertension { } 3. Diabetes { } 4. Tuberculosis { } 5. Other. Specify----
5. When were you diagnosed of this condition?
6. Less than a year ago { } 2. 1 to 5 years ago { } 3. 5 to 10 years ago { } 4. More than 10years ago { }

**COMPLIANCE TO MEDICATION REGIME**

1. How many different medications do you take for your condition?
2. One { } 2. Two { } 3. Three { } 4. Four { } 5. More than four { }
3. How often are your medications taken in a day?

1. Once daily [ ] 2. Twice daily [ ] 3. Thrice daily [ ] 4. Others…state…

1. What is the route of administration of the drug?
2. Orally [ ] 2. By injection[ ]
3. How many tablets/capsules do you take in a day, if your route of administration is oral?

1.Less than 5[ ] 2.Between 5 and 10[ ] 3.More than 10[ ]

1. Do you always adhere to your doctor’s instructions concerning how your medications should be taken? 1. Yes [ ] 2. No [ ]
2. Have you noticed any side effect(s) of the drug?

1. Yes [ ] 2. No [ ]

1. If yes please state the side effect(s)

_____________________________________________________________________

1. Are you able to tolerate the side effects of your medications? 1. Yes [ ] 2. No[ ]
2. Do you take any drug outside your doctor’s prescription e.g. Herbal preparations?

1. Yes [ ] 2. No [ ]

1. If yes, how does it influence your compliance to your medications?

_____________________________________________________________________

1. Do you have difficulty remembering medication instructions?

1. Yes [ ] 2. No [ ]

1. Do you think your medication(s) is effective?

1. Yes [ ] 2. No [ ]

1. How often do you forget to take your medications?

1. Daily [ ] 2. Frequently [ ] 3. Rarely [ ] 4. Never[ ]

1. Are you aware of the complications that can arise from non-compliance to your doctor’s prescription? 1. Yes [ ] 2. No [ ]
2. In what ways are you reminded to be compliant with your medications.

1. Doctor’s advice [ ] 2. Advice from other health workers [ ] 3. Radio or TV programmes [ ] 4. Friends or family [ ]

**C. THE INFLUENCE OF COST ON COMPLIANCE**

1. Is your medication covered by the NHIS?

1. Yes [ ] 2. No [ ]

1. If no, how do you pay for your drugs?
2. From my income [ ] 2. With support of family members [ ] 3. NGO sponsored [ ] 4. Medication is free [ ] 5. Others. Specify____________________
3. What is the cost of your medications? Name and cost____________________________
4. Have you ever missed your medication for a period of time because of cost of medication?
    1. Yes [ ] 2. No[ ]
